# Exome sequencing in 51 early onset non‐familial CRC cases

**DOI:** 10.1002/mgg3.605

**Published:** 2019-02-27

**Authors:** Jessada Thutkawkorapin, Annika Lindblom, Emma Tham

**Affiliations:** ^1^ Department of Molecular Medicine and Surgery Karolinska Institutet Stockholm Sweden; ^2^ Department of Molecular Medicine and Surgery Karolinska Institutet and Department of Clinical Genetics Karolinska University Hospital Stockholm Sweden

**Keywords:** colorectal cancer, early onset, exome sequencing, non‐familial

## Abstract

**Background:**

Colorectal cancer (CRC) cases with an age of onset <40 years suggests a germline genetic cause. In total, 51 simplex cases were included to test the hypothesis of CRC as a mendelian trait caused by either heterozygous autosomal dominant or bi‐allelic autosomal recessive pathogenic variants.

**Methods:**

The cohort was whole exome sequenced (WES) at 100× coverage. Both a dominant‐ and recessive model were used for searching predisposing genetic factors. In addition, we assayed recessive variants of potential moderate risk that were enriched in our young‐onset CRC cohort. Variants were filtered using a candidate cancer gene list or by selecting variants more likely to be pathogenic based on variant type (e.g., loss‐of‐function) or allele frequency.

**Results:**

We identified one pathogenic variant in *PTEN* in a patient subsequently confirmed to have a hereditary hamartoma tumor syndrome (Cowden syndrome) and one patient with a pathogenic heterozygous variant in *PMS2* that was originally not identified by WES due to low quality reads resulting from pseudogenes. In addition, we identified three heterozygous candidate missense variants in known cancer susceptibility genes (*BMPR1A*,*BRIP1*, and *SRC)*, three truncating variants in possibly novel cancer genes (*CLSPN*,*SEC24B**, **SSH2*) and four candidate missense variants in *ACACA**, **NR2C2**, **INPP4A,* and *DIDO1**.*** We also identify five possible autosomal recessive candidate genes: *ATP10B*,*PKHD1*,*UGGT2*,*MYH13*,*TFF3*.

**Conclusion:**

Two clear pathogenic variants were identified in patients that had not been identified clinically. Thus, the chance of detecting a hereditary cancer syndrome in patients with CRC at young age but without family history is 2/51 (4%) and therefore the clinical benefit of genetic testing in this patient group is low. Of note, using stringent filtering, we have identified a total of ten candidate heterozygous variants and five possibly biallelic autosomal recessive candidate genes that warrant further study.

## 1 INTRODUCTION

Colorectal cancer (CRC) is the third most common cancer diagnosed in Sweden (“Cancer fact sheet. World Health Organization”, 2010). In Sweden, familial CRC represents approximately 13% of all CRC cases (Frank, Sundquist, Yu, Hemminki, & Hemminki, 2017), while the cases with age of onset <40 years account for 1.4% (“Statistics on Cancer Incidence in Sweden 2016,” 2017). Known CRC susceptibility genes are responsible for 3.5% of all cases (Aaltonen, Johns, Jarvinen, Mecklin, & Houlston, 2007). In a study with over 10,000 CRC cases, Lynch syndrome was responsible for 3.1% overall (Moreira et al., 2012) and in 11/140 (8%) of CRC before 50 years (Giraldez et al., 2010). The same study detected 4/140 (3%) with bi‐allelic *MUTYH* mutations. Factors other than monogenic variants that contribute to cancer development are somatic genetic and epigenetic aberrations, combinations of low‐ and moderate‐risk genetic variation, individual life style (Lichtenstein et al., 2000), chronic bowel inflammation (Beaugerie & Itzkowitz, 2015), and environmental factors (Bodmer & Bonilla, 2008). However, CRC development before the age of 40 years and without a family history of CRC is unusual, suggesting either a de novo monogenic factor in a high‐risk autosomal dominant cancer gene, an autosomal dominant syndrome with decreased penetrance or a high‐risk autosomal recessive gene, or complex disease.

Here, we used exome sequencing of 51 simplex CRC cases with age of onset <40 years in order to search for novel monogenic cancer genes that cause a rare autosomal dominant or autosomal recessive colorectal cancer syndrome. In addition, we looked for moderate recessive risk variants that were enriched in our young‐onset CRC cohort.

## 2 MATERIALS AND METHODS

### 2.1 Ethical compliance

All patients gave written informed consent in accordance with Swedish legislation (2003:460) and the study was approved by the Regional Research Ethics Committee, Dnr: 2002‐20489, 2008/125‐2031/2, and 2014/1326‐32.

### 2.2 Early‐onset CRC cohort

CRC patients with age of onset before 40 years and without a family history of CRC were recruited through the Department of Clinical Genetics, Karolinska University Hospital Solna (Sweden) (*n* = 17) or recruited in a nationwide study, the Swedish Low‐risk Colorectal Cancer Study (*n* = 34). The latter consisted of more than 3,300 consecutive patients operated on for CRC in 14 hospitals in and around Stockholm and Uppsala between 2004 and 2009. All patients gave written informed consent to participate in the study and DNA was isolated from peripheral blood. The cancer diagnosis and histopathology were verified in all cases through medical records (Ghazi, 2010). For the present study, all simplex CRC cases diagnosed before 40 years were selected.

### 2.3 Familial Breast cancer (BRC) cohort used as a comparison group in autosomal recessive gene analysis

The familial BRC cohort was defined as individuals from families where at least two first or second‐degree relatives were affected with BRC. The families were recruited through the Department of Clinical Genetics, Karolinska University Hospital Solna (Sweden).

### 2.4 Whole exome sequencing of early onset CRC and familial BRC samples

DNA was quantified using a Qubit Fluorometer (Life Technologies, USA). Sequencing libraries were prepared according to the TruSeq DNA Sample Preparation Kit EUC 15005180 or EUC 15026489 (Illumina, USA). Briefly, 1–1.5 μg of genomic DNA was fragmented using the Covaris 400 bp protocol (Covaris, Inc., USA). After fragmentation, all samples were subjected to end‐repair, A‐tailing, and adaptor ligation of Illumina Multiplexing PE adaptors. An additional gel‐based size selection step was performed and the adapter‐ligated fragments were subsequently enriched by PCR followed by purification using Agencourt AMPure Beads (Beckman Coulter, Sweden). Exome capture was performed by pre‐pooling equimolar amounts and performing enrichment in 5‐ or 6‐plex reactions according to the TruSeq Exome Enrichment Kit Protocol (EUC 15013230). Library size was checked on a Bioanalyzer High Sensitivity DNA chip (Agilent Technologies, Sweden) while concentration was calculated by quantitative PCR. The pooled DNA libraries were clustered on a cBot instrument (Illumina) using the TruSeq PE Cluster Kit v3. Paired‐end sequencing was performed for 100 cycles using a HiSeq 2000 instrument (Illumina) with TruSeq SBS Chemistry v3, according to the manufacturer's protocol. Base calling was performed with RTA (1.12.4.2 or 1.13.48) and the resulting BCL files were filtered, de‐multiplexed, and converted to FASTQ format using CASAVA 1.7 or 1.8 (Illumina). The sequencing was performed at an average coverage of 100x.

### 2.5 Bioinformatics workflow

Sequencing reads were aligned to the reference genome GRCh37 using BWA (H. Li & Durbin, 2010). Aligned reads were sorted and PCR‐duplicated reads were removed using Picard (http://broadinstitute.github.io/picard/). The calculation of mapping and enrichment statistics were done with Picard and GATK (McKenna et al., 2010). Variants were called using GATK by following the best practice procedure implemented at the Broad Institute (DePristo et al., 2011). Variant Quality Score Recalibration from GATK was used for quality control of the variants. Variant annotation was done by ANNOVAR (Wang, Li, & Hakonarson, 2010). The annotated information includes RefSeq gene annotation (O'Leary et al., 2016), dbSNP rs number (Sherry et al., 2001), COSMIC (Forbes et al., 2017), ClinVar (Landrum et al., 2018), SPIDEX (Xiong et al., 2015), ExAC conservative constraint (Lek et al., 2016), UniProt (Chen, Huang, & Wu, 2017). Background allele frequencies are from SweGen (Ameur et al., 2017), ExAC (Lek et al., 2016), gnomAD (Lek et al., 2016), and 1000 Genomes Project allele frequencies (1000 Genomes Project Consortium et al., 2012). In silico predictors used for predicting pathogenic effects include SIFT (Kumar, Henikoff, & Ng, 2009), PolyPhen2 (Adzhubei et al., 2010), Phylop (Cooper et al., 2005), LRT (Chun & Fay, 2009), Mutation Taster (Schwarz, Rodelsperger, Schuelke, & Seelow, 2010), Mutation Assessor (Reva, Antipin, & Sander, 2011), FATHMM (Shihab et al., 2015), GERP++ (Davydov et al., 2010), and CADD (Kircher et al., 2014).

### 2.6 Allele frequency database

In this study, we have used maximum minor allele frequency (MMAF) from 21 populations (SweGen (Ameur et al., 2017), 1000G (The 1000 Genomes Project Consortium, 2012), ExAC (Lek et al., 2016), gnomAD (Lek et al., 2016), 200Danes (Y. Li et al., 2010), and 249Swedes (http://neotek.scilifelab.se/hbvdb/)) to identify common variants.

### 2.7 Analysis workflows

#### 2.7.1 Rare autosomal dominant and autosomal recessive analysis in cancer susceptibility gene list

**Figure 1:**
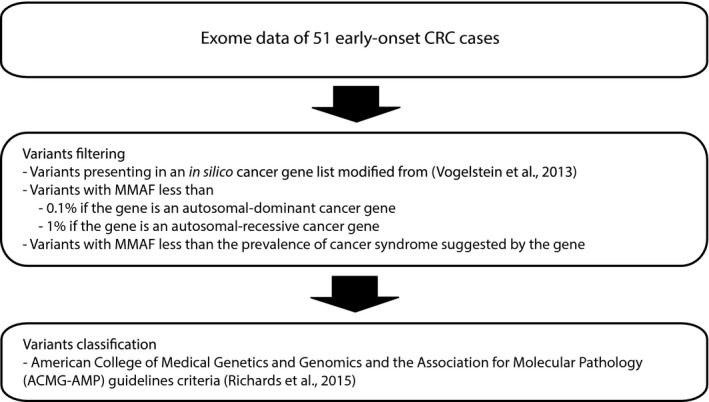
Autosomal dominant and autosomal recessive analysis in cancer susceptibility gene list

To search for causative mutations in autosomal dominant‐ or autosomal recessive cancer susceptibility genes, we first used an in silico cancer gene list modified from (Vogelstein et al., 2013) in the analysis of 51 early‐onset CRC samples. The gene list contains 244 known cancer‐related genes (known somatic cancer driver genes as well as hereditary cancer genes). The candidate variants were primarily selected by filtering splicing and non‐silent variants with MMAF <1% in autosomal‐recessive cancer genes and <0.1% in autosomal‐dominant cancer genes. The variants with MMAF higher than the prevalence of cancer syndrome suggested by the gene were manually excluded. The resulting candidate variants were classified as of uncertain significance, likely pathogenic, or pathogenic using American College of Medical Genetics and Genomics and the Association for Molecular Pathology (ACMG‐AMP) guidelines criteria (Richards et al., 2015) (Figure 1). The candidate variants were then confirmed by Sanger sequencing. Manual analysis of poor quality *PMS2* variants using the sequencing file (BAM) was performed.

#### 2.7.2 Frameshift‐, nonsense‐, and splice variants analysis

**Figure 2:**
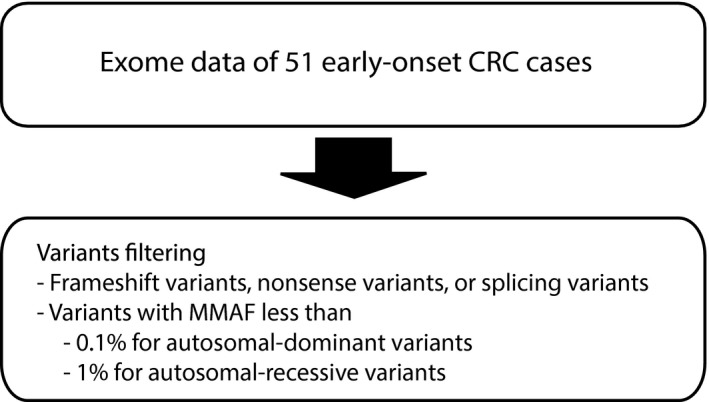
Frameshift‐, nonsense‐, and splice variants analysis

We selected all frameshift‐, nonsense‐, and splice variants in the exome which have MMAF < 0.1% (for heterozygous variants) and <1% (for recessive variants) (Figure 2). The candidate variants were then confirmed by Sanger sequencing.

#### 2.7.3 Analysis of missense variants

**Figure 3:**
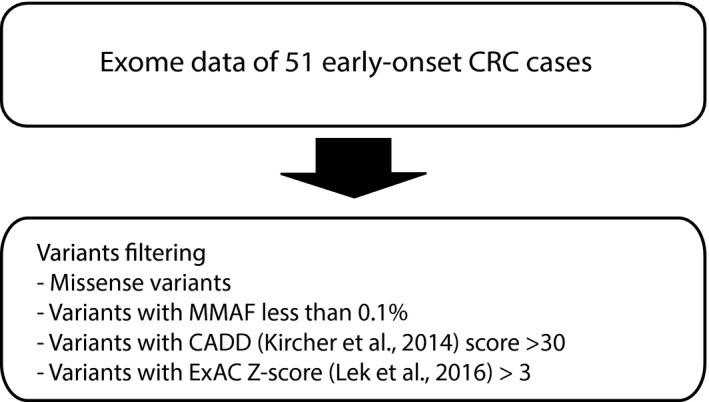
Analysis of missense variants

In this analysis, we selected all missense variants in the exomes of the 51 CRC patients with MMAF < 0.1% in all public databases. Then, we grouped the variants using CADD score (Kircher et al., 2014) (“more than 20”, “more than 25”, and “more than 30”) and ExAC missense *Z*‐score (Lek et al., 2016) (“less than or equal to 3” and “more than 3”) (Figure 3).

#### 2.7.4 Autosomal recessive genes analysis (rare monogenic and less common risk genes)

**Figure 4:**
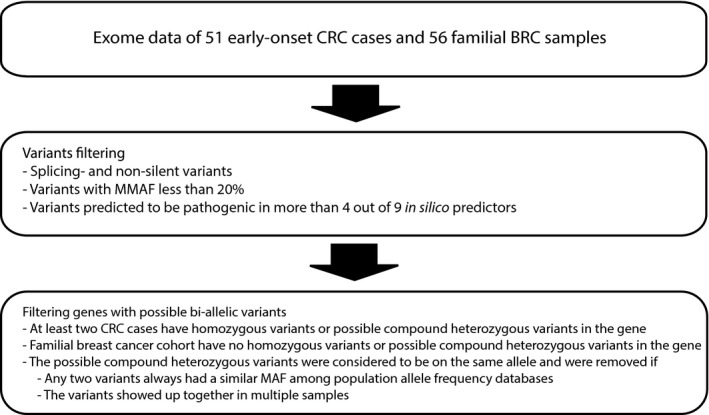
Autosomal recessive genes analysis

To pinpoint autosomal recessive genes predisposing to early‐onset colorectal cancer, we hypothesized that the patients inherited biallelic risk variants, one from each parent in a homozygous or compound heterozygous state. A gene was assumed to be associated with an increased risk of CRC if the number of cases with the possible biallelic variants was more prevalent than in a control population. Due to an absence of individual genotyping data of a normal population, we used a cohort of familial breast cancer processed in the same way as the CRC samples as a comparison group, assuming that the possible biallelic variants predisposing to early‐onset colorectal cancer are not associated with familial breast cancer. With such a hypothesis, we searched for possible biallelic variants in the exome in our 51 early‐onset CRC cases with 56 familial BRC as a comparison group. During the first step of filtering, genes with splicing and non‐silent variants, with a MMAF < 20% and predicted to be pathogenic by more than 4 out of 9 in silico effect predictors (SIFT (Kumar et al., 2009), PolyPhen2 HDIV (Adzhubei et al., 2010), PolyPhen2 HVAR (Adzhubei et al., 2010), Phylop (Cooper et al., 2005), LRT (Chun & Fay, 2009), Mutation Taster (Schwarz et al., 2010), Mutation Assessor (Reva et al., 2011), FATHMM (Shihab et al., 2015), GERP++ (Davydov et al., 2010)), were selected. The next filtering step was to select genes with possible biallelic variants (possible compound heterozygous or homozygous) in at least two CRC cases and where no possible compound or homozygous case was found among the familial breast cancer cohort. If any two variants always had a similar MAF among population allele frequency databases or the variants showed up together in multiple samples, the variants were considered likely to be on the same allele and were excluded (Figure 4). The variants were prioritized based on MMAF and the observed frequency in CRC compared to the statistical likelihood of occurring together (computed by multiplying the MMAF of the two alleles).

### 2.8 Sanger sequencing

Primer pairs were designed using Primer3Plus (http://www.bioinformatics.nl/primer3plus) to amplify the candidate pathogenic variants suggested by exome sequencing data. PCR was performed in 25 μl reaction volumes containing AmpliTaq Gold (Life Technologies, Carlsbad, CA), 1.25 μg DNA and 10 μmol l^−1^ of primers. Excess single‐stranded DNA, primers, and dNTPs were removed using ExoSAP. The sequences were visualized and analyzed using CodonCode Aligner (http://www.codoncode.com).

### 2.9 Database submission of novel variants

Relevant variant information has been uploaded to the Leiden Open Variation Database (LOVD), http://www.lovd.nl/3.0

## 3 RESULTS

We first searched for variants in known cancer susceptibility genes (Figure 1), resulting in eight heterozygous variants in eight genes (Table 1, Figure 1). Among them, the variant in *PTEN*, p.Ser59Ter, has been reported to be pathogenic in ClinVar (Landrum et al., 2018) and had been reported as a somatic mutation 15 times in the TCGA dataset in various tumor types in cBioportal (Cerami et al., 2012; Gao et al., 2013). The patient was subsequently examined at the Department of Clinical Genetics at an age of 50 years and found to have macrocephaly (OFC: 65 cm), papillomatous lesions on hands and oral mucosa, intellectual disability (IQ = 84), and hamartomatous intestinal polyps. He was sent to surveillance of the thyroid according to clinical guidelines and multinodular goiter was detected. In all, these findings confirmed the clinical diagnosis of Cowden syndrome/Hamartoma tumor syndrome (MIM #601728). His parents were both 23 years of age when he was born and neither had had any cancer. Genetic counseling was offered to his family members. Unfortunately, no family members have contacted us and we have therefore not been able to perform genetic testing of the parents/siblings in order to confirm a de novo or familial variant. There were six candidate missense variants in the following cancer genes: *MSH2*,* APC*,* PTPN12*,* BMPR1A*,* POLE,* and *SRC,* and one inframe duplication in *BRIP1*. All were classified as variants of unknown significance according to ACMG criteria (Table 1). All of these genes are expressed in colon tissue (www.genecards.org), but none of these specific variants have been reported as somatic variants in 3,473 colorectal cancer samples or in any other tumor types in cBioPortal. After our study, immunohistochemistry of mismatch repair genes was performed as part of another research project on one patient with CRC at 37 years. His tumor showed loss of PMS2 protein. Targeted sequencing of *PMS2* (based on a clinical nested PCR approach) detected a NM_000535.5:c.2113G>A, p.Glu705Lys variant that is known to be pathogenic and has been reported as causative in many families, even though the INSIGHT expert panel have interpreted it as a variant of unknown significance (ClinVar). Thereafter, we manually checked the sequencing files of the other patients for poor quality *PMS2* variants and no other possible pathogenic variant in *PMS2* was found. No homozygous/compound heterozygous variants were found among the 244 cancer genes.

**Table 1:** Candidate variants from cancer‐gene‐list study

Gene name	Protein change	cDNA change	Protein domain^a^	MMAF^b^	ClinVar	CADD^c^	Samples
**PTEN**	**NP_000305.3:p.Ser59Ter**	**NM_000314.3:c.176C>A**	Phosphatase tensin‐type	**NA**	Pathogenic	50	**48‐07LF**
APC	NP_000029.4:p.Asn944Asp	NM_000038.4:c.2830A>G	No special domain	0.05%	VUS	10.93	329‐09F
MSH2	NP_000242.2:p.Val722Phe	NM_000251.2:c.2164G>T	No special domain	0.02%	VUS	34	1068‐05o
PTPN12	NP_002826.6:p.Asp26Glu	NM_002835.6:c.78C>G	No special domain	0.01%	NA	11.9	323‐09F
POLE	NP_006222.5:p.Pro13Ser	NM_006231.5:c.37C>T	No special domain	0.01%	NA	10.64	Co‐121
BMPR1A	NP_004320.1:p.Ala293Val	NM_004329.3:c.878C>T	Cytoplasmic and protein kinase domain	NA	NA	27.2	Co‐286
BRIP1	NP_114432.2:p.Asn1161_Asp1163dup	NM_032043.2:c.3481_3489dup	No special domain	NA	NA	NA	4283‐13D
SRC	NP_938033.4:p.Gln112His	NM_198291.4:c.336G>C	SH3 domain	NA	NA	12.22	59‐04o

VUS, variant of unknown significance.

Bold font indicates a known pathogenic variant based on it being a nonsense mutation, not previously reported in healthy individuals, but reported in patients with Cowden syndrome.

a UniProt protein domain.

b Maximum minor allele frequency from ExAC ALL, ExAC NFE, ExAC AFR, ExAC AMR, ExAC EAS, ExAC FIN, ExAC SAS, ExAC OTH, gnomAD ALL, gnomAD AFR, gnomAD AMR, gnomAD ASJ, gnomAD EAS, gnomAD FIN, gnomAD NFE, gnomAD OTH, 1000G ALL, 1000G EUR, SweGen 249DANES, 249SWEDES.

c CADD score.

In addition to the cancer gene list, we also performed an analysis on frameshift‐, nonsense‐, and splice variants in the exome (Figure 2). After Sanger verification, apart from the variant in *PTEN*, there were 10 possible candidate variants from 10 genes (*CLSPN, CELSR2, ADAM17, BIRC6, SEC24B, RBM27, PPARGC1B, NCOA7, SSH2,* and *MYO9B)* (1 frameshift deletion, 7 nonsense‐, and 2 splice variants) (Table 2). All these genes are expressed in the normal colon tissue (genecards). None of the variants were found in cBioPortal.

**Table 2:** Candidate truncating variants

Gene name	Protein change	cDNA change	Protein domain^a^	MMAF^b^	Samples
**PTEN**	**NP_000305.3:p.Ser59Ter**	**NM_000314.3:c.176C>A**	Phosphatase tensin‐type	NA	48‐07LF
CLSPN	NP_071394.5:p.Arg1040Ter	NM_022111.5:c.3118C>T	No special domain	NA	2750‐13D
CELSR2	NP_001399.3:p.Asp843IlefsTer65	NM_001408.3:c.2527del	Cadherin 9	NA	4270‐11D
ADAM17	NP_003174.3:p.Gln471Ter	NM_003183.3:c.1411C>T	Peptidase M12B	NA	760‐04o
BIRC6	NA	NM_016252.2:c.12811‐1G>A	No special domain	0.05%	1307‐09D
SEC24B	NP_006314.1:p.Arg307Ter	NM_006323.2:c.919C>T	No special domain	NA	Co‐286
RBM27	NA	NM_018989.1:c.590‐2A>C	Arg‐rich domain	0.05%	4735‐11D
PPARGC1B	NP_573570.5:p.Trp328Ter	NM_133263.5:c.983G>A	No special domain	NA	294‐07LF
NCOA7	NP_001186548.3:p.Arg913Ter	NM_001199619.3:c.2737C>T	TLD domain	0.02%	664‐08F
SSH2	NP_203747.1:p.Tyr1292Ter	NM_033389.1:c.3876C>G	No special domain	NA	49‐06o
MYO9B	NP_004136.1:p.Cys1646Ter	NM_004145.1:c.4938C>A	Phorbol‐ester/DAG‐type	NA	Co‐489

All the variants in this table are heterozygous and were not reported in ClinVar. FIN, ExAC SAS, ExAC OTH, gnomAD ALL, gnomAD AFR, gnomAD AMR, gnomAD ASJ, gnomAD EAS, gnomAD FIN, gnomAD NFE, gnomAD OTH, 1000G ALL, 1000G EUR, SweGen 249DANES, 249SWEDES.

Bold font indicates a known pathogenic variant based on it being a nonsense mutation, not previously reported in healthy individuals, but reported in patients with Cowden syndrome.

a UniProt protein domain.

b Maximum minor allele frequency from ExAC ALL, ExAC NFE, ExAC AFR, ExAC AMR, ExAC EAS, ExAC.

In missense variants analysis (Figure 3), there were in total 3,800 missense variants with MMAF < 0.1% in all public databases. In order to select candidate variants, we filtered the variants with CADD score (Kircher et al., 2014) more than 20 (*n* = 2301) (Supporting Information, Table S1). Among them, there were 248 variants in genes in which the normal population has fewer missense variants than expected, ExAC missense *Z*‐score > 3 (Lek et al., 2016), (Supporting Information, Table S2), suggesting that missense variants might be detrimental to the normal gene function. If we instead categorize the 2,301 variants based on higher CADD score, 1,140 variants had a CADD score >25 and 367 variants had a CADD score of more than 30 (Supporting Information, Table S2). 669/2301 variants had never been reported in a normal population and, of those with CADD >30, 82 had never been reported and eight of these were in a gene where missense variants were expected to be deleterious based on the conservative constraint, ExAC missense *Z*‐score (Lek et al., 2016) resulting in the following candidate gene list: *ACACA, KIAA1109, GPHN, NR2C2, GPR25, ZNF462, INPP4A,* and *DIDO1* (Table 3).

**Table 3:** Candidate missense variants from whole exome sequencing

Gene name	Protein change	Protein domain^a^	MMAF^b^	Mis Z^c^	CADD^d^	Samples
KIAA1109	NM_015312:p.S3578F	No special domain	0.0001	1.210177	28.1	711‐14D
GPR25	NM_005298:p.Arg228Cys	Cytoplasmic domain	0	3.4183516	35	4270‐11D
INPP4A	NM_001134224:p.Arg901His	No special domain	0	3.1907657	34	295‐08F
NR2C2	NM_003298:p.Arg411His	Nuclear receptor (NR) ligand‐binding (LBD) domain	0	3.481554	35	329‐09F
ZNF462	NM_021224:p.Arg2068Gln	In zinc finger: C2H2‐type 20	0	3.2241783	35	679‐05o
GPHN	NM_020806:p.Gly472Asp	No special domain	0	3.571934	32	376‐05o
ACACA	NM_198834:p.Arg2208Gln	No special domain	0.0000899	7.5240764	35	1307‐09D
DIDO1	NM_033081:p.Arg1145His	No special domain	0	3.0966303	35	1068‐05o

a UniProt protein domain.

b Maximum minor allele frequency from ExAC ALL, ExAC NFE, ExAC AFR, ExAC AMR, ExAC EAS, ExAC FIN, ExAC SAS, ExAC OTH, gnomAD ALL, gnomAD AFR, gnomAD AMR, gnomAD ASJ, gnomAD EAS, gnomAD FIN, gnomAD NFE, gnomAD OTH, 1000G ALL, 1000G EUR, SWEGEN 249DANES, 249SWEDES.

c ExAC missense Z Score.

d Combined Annotation Dependent Depletion (CADD) score.

In the autosomal recessive gene analysis (Figure 4), there were 16 genes where at least two CRC cases had homozygous or possible compound heterozygous variants and none in the breast cancer cohort that we used as a comparison group (Table 4). Six genes (*ATP10B*,* PKHD1*,* PTPRQ*,* UGGT2*,* MYH13*,* TFF3*) had variants with MMAF <5%, which were observed together in CRC cases twenty times more than expected by the computed likelihood. Therefore, these six genes are more likely to be candidate autosomal recessive risk factors.

**Table 4:** Sixteen candidate autosomal recessive genes where at least two CRC cases had homozygous or possible compound heterozygous variants and none in the breast cancer cohort that we used as a comparison group

Gene name	Protein change	cDNA change	Coordinate^a^	Likelihood^b^	obs gnomAD^c^	obs CRC^d^	MMAF^e^	CRC^f^	BRC^g^	Patient1^h^	Patient2^i^
**ATP10B**	NP_079429.2:p.Asn865Lys	NM_025153.2:c.2595C>A	5:g.160042903G>T				0.0430	0.0294	NA	48‐07LF (het)	569‐04o (het)
	NP_079429.2:p.Gly393Trp	NM_025153.2:c.1177G>T	5:g.160061565C>A	0.0008	NA	0.0400	0.0199	0.0196	0.0179	48‐07LF (het)	569‐04o (het)
**PKHD1**	NP_619639.3:p.Thr2869Lys	NM_138694.3:c.8606C>A	6:g.51637536G>T				0.0237	0.0490	0.0179	566‐04o (het)	
	NP_619639.3:p.Ser1833Leu	NM_138694.3:c.5498C>T	6:g.51882310G>A	0.0000	NA	0.0200	0.0005	0.0098	NA	566‐04o (het)	
	NP_619639.3:p.Leu1709Phe	NM_138694.3:c.5125C>T	6:g.51889483G>A				0.0053	0.0098	NA		1199‐05o (het)
	NP_619639.3:p.Cys843Ser	NM_138694.3:c.2527T>A	6:g.51910867A>T	0.0000	NA	0.0200	0.0000	0.0098	NA		1199‐05o (het)
**PTPRQ**	NP_001138498.1:p.Pro98Leu	NM_001145026.1:c.293C>T	12:g.80839400C>T				0.0015	0.0098	NA	4773‐11D (het)	
	NP_001138498.1:p.Gln425Glu	NM_001145026.1:c.831A>G	12:g.80878310A>G				0.0625	0.0294	NA		386‐04o (het)
	NP_001138498.1:p.Thr1967Met	NM_001145026.1:c.5458C>T	12:g.81028849C>T	0.0001	NA	0.0200	0.0012	0.0098	NA		386‐04o (het)
	NP_001138498.1:p.Ile2207Val	NM_001145026.1:c.6177A>G	12:g.81066976A>G	0.0001	NA	0.0200	0.0485	0.0098	NA	4773‐11D (het)	
**UGGT2**	NP_064506.3:p.Asn1268Tyr	NM_020121.3:c.3802A>T	13:g.96511868T>A	0.0002	NA	0.0200	0.0310	0.0392	0.0089	Co‐1524 (het)	
	NP_064506.3:p.Arg480His	NM_020121.3:c.1439G>A	13:g.96601605C>T	0.0006	0.0002	0.0200	0.0078	0.0294	NA	Co‐1524 (het)	61‐09F (hom)
**MYH13**	NP_003793.2:p.Arg1438Cys	NM_003802.2:c.4312C>T	17:g.10215944G>A				0.0169	0.0196	0.0179	784‐05o (het)	
	NP_003793.2:p.Ala1128Val	NM_003802.2:c.3383C>T	17:g.10222462G>A				0.0149	0.0098	0.0089		Co‐286 (het)
	NP_003793.2:p.Lys807del	NM_003802.2:c.2420_2422del	17:g.10233717_10233719del	0.0001	NA	0.0200	0.0065	0.0098	0.0268		Co‐286 (het)
	NP_003793.2:p.Gly701Arg	NM_003802.2:c.2101G>A	17:g.10236464C>T	0.0006	NA	0.0200	0.0331	0.0392	NA	784‐05o (het)	
**TFF3**	NP_003217.2:p.Val116Met	NM_003226.2:c.346G>A	21:g.43733628C>T	0.0001	0.0001	0.0400	0.0089	0.0568	NA	1205‐05o (hom)	760‐04o (hom)
PCDHGB4	NP_115269.1:p.Pro15_Val16del	NM_032098.1:c.44_49del	5:g.140767492_140767497del	0.0360	0.0180	0.0400	0.1905	0.0784	0.0909	294‐07LF (hom)	Co‐286 (hom)
BCLAF1	NP_001070908.1:p.Glu401Ter	NM_001077440.1:c.1201G>T	6:g.136597456C>A				0.0393	0.0196	NA	2014‐04018 (het)	91‐04o (het)
	NP_001070908.1:p.Arg45Met	NM_001077441.1:c.134G>T	6:g.136599885C>A	0.0030	NA	0.0400	0.1049	0.0588	0.0893	2014‐04018 (het)	91‐04o (het)
ZNRF2	NP_667339.3:p.Asp123dup	NM_147128.3:c.368_370dup	7:g.30325341_30325343dup	0.0027	0.0006	0.0400	0.0526	0.0588	0.0577	813‐06o (hom)	91‐04o (hom)
AGPAT2	NP_006403.3:p.Val18dup	NM_006412.3:c.50_52dup	9:g.139581758_139581760dup	0.0110	0.0086	0.0400	0.1054	0.1765	0.0545	749‐08F (hom)	760‐04o (hom)
MRGPRX3	NP_473372.3:p.Trp307Ter	NM_054031.3:c.920G>A	11:g.18159669G>A	0.0070	0.0060	0.0400	0.0855	0.1275	0.0893	1307‐09D (hom)	784‐05o (hom)
TPSG1	NP_036599.3:p.Arg194Trp	NM_012467.3:c.580C>T	16:g.1272273G>A	0.0016	NA	0.0200	0.0132	0.0098	NA		4270‐11D (het)
	NP_036599.3:p.Ser138Phe	NM_012467.3:c.413C>T	16:g.1272750G>A	0.0150	0.0090	0.0200	0.1225	0.0784	0.0446	1094‐08F (hom)	4270‐11D (het)
	NP_036599.3:p.Thr75Lys	NM_012467.3:c.224C>A	16:g.1273444G>T	0.0240	0.0070	0.0200	0.1546	0.0196	0.0364		711‐14D (hom)
FBXW10	NP_001254514.1:p.Arg275Leu	NM_001267585.1:c.824G>T	17:g.18653188G>T	0.0120	0.0070	0.0200	0.1134	0.0882	0.0804	Co‐1190 (hom)	1199‐05o (het)
	NP_001254514.1:p.Arg607Cys	NM_001267585.1:c.1819C>T	17:g.18671961C>T	0.0150	NA	0.0200	0.1320	0.0686	0.0268		1199‐05o (het)
ZNF730	NP_001264332.1:p.Thr196Ala	NM_001277403.1:c.586A>G	19:g.23328432A>G				0.0921	0.0196	0.0446		771‐08F (het)
	NP_001264332.1:p.Ser296Phe	NM_001277403.1:c.887C>T	19:g.23328733C>T	0.0060	NA	0.0200	0.0649	0.0392	0.0446	711‐14D (het)	771‐08F (het)
	NP_001264332.1:p.Ile479AsnfsTer	NM_001277403.1:c.1429_1430insA	19:g.23329275_23329276insA	0.0004	NA	0.0200	0.0065	0.0098	NA	711‐14D (het)	
TARM1	NP_001129158.1:p.Arg111His	NM_001135686.1:c.332G>A	19:g.54578105C>T	0.0100	0.0200	0.0400	0.0979	0.1176	0.0638	5053‐10D (hom)	Co‐489 (hom)
RFPL1	NP_066306.2:p.Gln243Ter	NM_021026.2:c.727C>T	22:g.29837884C>T	0.0370	0.0300	0.0400	0.1931	0.1863	0.125	258‐06o (hom)	4283‐13D (hom)

Six genes (ATP10B, PKHD1, PTPRQ, UGGT2, MYH13 and TFF3) had variants, which were observed together in CRC cases twenty times more than expected by the computed likelihood.

Bold font indicates variants that are rare in the normal population and have a low liekliheood of occuring together and are therefore possible candidate variants.

a Genomics coordinate based on GRCh37.

b Likelyhood to observe homozygous‐ or possible compound heterozygous variants based on MMAF of the two alleles.

c Frequency of observed homozygous‐ or possible compound heterozygous variants in 51 early onset colorectal cancer samples.

d Maximum obsevered frequency of homozygous samples in gnomAD ALL, gnomAD AFR, gnomAD AMR, gnomAD ASJ, gnomAD EAS, gnomAD FIN, gnomAD NFE, gnomAD OTH.

e Maximum minor allele frequency from ExAC ALL, ExAC NFE, ExAC AFR, ExAC AMR, ExAC EAS, ExAC FIN, ExAC SAS, ExAC OTH, gnomAD ALL, gnomAD AFR, gnomAD AMR, gnomAD ASJ, gnomAD EAS, gnomAD FIN, gnomAD NFE, gnomAD OTH, 1000G ALL, 1000G EUR, SweGen 249DANES, 249SWEDES.

f Allele frequency in 51 early onset colorectal cancer samples.

g Allele frequency in 56 breast cancer samples.

h Patients with homozygous‐ and possible compound heterozygous variants.

## 4 DISCUSSION

Since onset of colorectal cancer before the age of 40 years is infrequent, genetic predisposition may be suspected. Our cohort of 51 simplex cases with early‐onset CRC tested two hypotheses: first a pathogenic variant in an autosomal dominant cancer gene; and second an autosomal recessive cancer syndrome.

In order to address the first hypothesis, we searched for heterozygous variants in genes known to be important in hereditary cancer or known somatic driver genes according to the classification by the Vogelstein group (Vogelstein et al., 2013) (Table 1). We found one pathogenic variant in *PTEN*, p.Ser59Ter, known to cause Cowden/hamartoma tumor syndrome and this syndrome could later be confirmed clinically in the patient and genetic counseling could be offered to his relatives. While the other variants (Table 1) suggested by the cancer gene list approach have an unknown clinical significance (Landrum et al., 2018), the genes are known to be cancer‐related. As we did not have access to tumor material or DNA from relatives, unfortunately further analyses to test the gene expression in tumors and/or segregation in the family could not be performed. One patient demonstrated loss of PMS2 protein in his tumor and harbored a pathogenic variant in exon 12 in *PMS2* that was detected by a clinical lab. This variant was initially missed by our WES due to poor quality in the mapping step as a result of the pseudogene *PMS2CL* that has a high homology to exon 9 and 11–15 (Takeda et al., 2014). Loss of function in *APC* gene leads to the development of adenomas, a precursor lesion to CRC, in familial adenomatous polyposis (MIM #175100). This condition occurs in <1/10,000, thus the p.Asn944Asp variant with a MMAF of 0.05% is also probably too common to be disease causing. *MSH2* is known to cause Lynch syndrome (MIM #120435). The incidence of Lynch syndrome has been estimated to be 1/660–1/2,000 (de la Chapelle, 2005) and approximately 40% of cases are caused by *MSH2* mutation (i.e., incidence of MSH2‐Lynch syndrome: 1/1,650–1/5,000) (Moller et al., 2017). In the ClinVar database, there are 850 pathogenic variants in *MSH2* and thus the p.Val722Phe *MSH2* variant with an incidence of 1/5,000 (0.02%) is probably too common to be disease‐causing. *PTPN12* is critical for the regulation of cell proliferation, differentiation, and neoplastic transformation (Takekawa et al., 1992) but is not associated with a known clinical cancer syndrome. *POLE* encodes the catalytic subunit of DNA polymerase epsilon and mutations cause susceptibility to CRC and other tumor types (MIM # 615083). Both of these genes are somatically mutated in CRC, but these variants were also present in 0.01% of a normal population and are likely too common to be cancer predisposition variants. The *BMPR1A, BRIP1* and *SRC* variants have never been reported in healthy individuals which makes them highly interesting. *BMPR1A* variants can cause juvenile intestinal polyposis (MIM #174900). However, the only clinical information we had available from the patient was microsatellite‐stable CRC at 35 years with no information regarding possible polyps. We cannot exclude or verify this potential diagnosis clinically. *BRIP1* has been shown to be a moderate‐risk ovarian cancer gene (Ramus et al., 2015; Weber‐Lassalle et al., 2018), most often caused by truncating variants (ClinVar). Two CRC patients with truncating *BRIP1* variants and either familial CRC or polyposis have recently been reported (Martin‐Morales et al., 2018; Rosenthal et al., 2018). However, the pathogenicity of our in‐frame *BRIP1* duplication is not known. *SRC*, encoding a tyrosine kinase receptor, is frequently implicated in cancer (Turro et al., 2016). It was suggested as a putative CRC gene based on proteomic analysis (Zhang et al., 2014) and may have a role in colon cancer progression (Irby et al., 1999).

In an attempt to search for novel cancer genes, we identified 10 truncating variants (in addition to the pathogenic *PTEN* variant) (Table 2) in genes which are not known to be susceptibility genes for cancer: *CLSPN, CELSR2, ADAM17, BIRC6, SEC24B, RBM27, PPARGC1B, NCOA7, SSH2,* and *MYO9B*. All these genes are expressed in colon tissue (genecards.org). Seven of these have never been reported in normal databases, supporting their role as rare risk variants. Three have a function highly related to cancer. *CLSPN* is involved in the DNA damage checkpoint (Chini & Chen, 2003) and is required for the activation of *CHK1* during a DNA replication checkpoint response (Kumagai & Dunphy, 2000). It is also involved in DNA replication and repair of DNA damage repair and may function as a tumor suppressor gene (Azenha, Lopes, & Martins, 2017). Nine somatic truncating mutations in *CLSPN* have been reported in CRC in cBioPortal including one at position 1139, that is, more distal to our variant. Therefore, this gene is the most likely candidate for loss‐of‐function mutations. The *CELSR2* protein was identified to contain epidermal growth factor‐like domains (Nakayama et al., 1998). It is important for the development of normal cilia and neuronal migration (Goffinet & Tissir, 2017). The expression of *CELSR2* protein was increased in the cytoplasm of breast cancer cells (Jiang et al., 2018), but its possible function in cancer is unclear. The *ADAM17* protein is a metalloprotease first identified as an enzyme that cleaves tumor necrosis factor‐alpha (Adrain, Zettl, Christova, Taylor, & Freeman, 2012; Black et al., 1997). Later it was found to have more than 80 substrates and is important for releasing ligands of *EGFR* from the membrane and thus activating *EGFR* (Zunke & Rose‐John, 2017). *ADAM17* is upregulated in colorectal cancer cells (Blanchot‐Jossic et al., 2005) and blocking *ADAM17* in mouse colorectal cancer xenografts inhibited tumor growth (Rios‐Doria et al., 2015). In humans, loss of both alleles of *ADAM17* can cause a rare neonatal autosomal recessive inflammatory bowel and skin disease (MIM #603639). As both *CELSR2* and *ADAM17* seem to have growth‐stimulating functions, the loss‐of‐function mutations are less likely candidates for cancer predisposition. The variants in *BIRC6, RBM27,* and *NCOA7* were found in 0.02%–0.05% of the reference population which reduces the likelihood of them being pathogenic. Of these, *BIRC6* exhibited a role in resistance to apoptosis (Chen et al., 1999; Hao et al., 2004). The gene has been suggested to be a prognosis predictor in colorectal cancer (Hu et al., 2015) and *BIRC6* mutations have been found in colorectal adenomas (Zhou et al., 2013) and are common in carcinomas (Wolff et al., 2018). *NCOA7/ERAP140* is a nuclear receptor coactivator that binds to the estrogen receptor among others. It has been implicated as a risk factor for breast cancer (Higginbotham et al., 2011) but has as yet no known association to CRC. It has been shown to be over‐expressed in oral squamous cell carcinoma where it promotes cell proliferation (Xie et al., 2016). Two genes have some association to cancer but not to CRC: *PPARGC1B* is a co‐activator for *ESR1* and variants in the gene have been associated with increased risk for estrogen‐positive breast cancer (Li et al., 2011). One study demonstrated that *MYO9B* was involved in the migration of prostate cancer cell lines and might be important for metastasis (Makowska, Hughes, White, Wells, & Peckham, 2015). To date, no studies have implicated *RBM27, SEC24B,* or *SSH2* in CRC or cancer development. As the variants in *SEC24B* and *SSH2* have never been reported in the normal population databases, they are also possible candidate genes.

In total, 3,800 rare missense variants were found in the exomes of the 51 young CRC cases. In order to try to reduce the number of candidates to consider, we filtered using CADD score > 20 which left 2301 missense variants (Supporting Information, Table S2). In order to pinpoint the candidate variants, we used even more stringent criteria (higher CADD, variants that were never previously reported and in genes that normally do not have many missense variants) giving a short‐list of eight variants. CADD represents multiple lines of in silico evidence of pathogenicity and can predict pathogenicity reasonably well, however, in silico tools (such as CADD and the ExAC missense *Z*‐score) will also miss clinically relevant pathogenic variants (van der Velde et al., 2015) and therefore this way of filtering is not optimal. In this way, we have probably excluded several variants that may have a pathogenic effect and in the future when many hundred thousand individuals have been sequenced in population databases, we may be able to filter solely on MMAF. Among the eight top candidate variants, one has been reported to be a possible tumor suppressor gene: *INPP4A*. *INPP4A* has been shown to be downregulated in pancreatic cancer and to inhibit cell proliferation and promote apoptosis in bladder and pancreatic cancer cells (Wang, Feng, Jiang, & Zuo, 2017; Wang, Wu, Huang, & Chen, 2018). Three have been described in cancer, but their function remains unclear. *ACACA* (acetyl‐CoA carboxylase or *ACC1*) catalyses the rate‐limiting reaction in the biogenesis of long‐chain fatty acids and is thus vital for cancer cell survival during hypoxia (Gao et al., 2016). Inhibition of *ACACA* can lead to either decreased cell proliferation (Jones et al., 2017; Singh, Yadav, Kumar, & Saini, 2015) or decreased apoptosis (Keenan et al., 2015) and increased risk of metastasis/tumor recurrence (in mice) (Rios Garcia et al., 2017). *NR2C2* can inhibit cancer initiation, but promote cancer progression (Lin et al., 2017). In colon cancer cell lines, *NR2C2* is required for cell survival and its inhibition induces cell death (McNew et al., 2016; Singh et al., 2012). *DIDO1* regulates embryonic stem cell renewal (Liu et al., 2014) and is upregulated in melanoma tissues and cell lines as well as colorectal tumors (Braig & Bosserhoff, 2013; Sillars‐Hardebol et al., 2012), although in the latter, there was no correlation to gene copy number and *DIDO1* was suggested to be a passenger on the common 20q duplication seen in CRC. The remaining four have no known cancer‐related function (*KIAA1109*,* GPHN*,* GPR25*,* ZNF462*). Thus, whole exome sequencing has demonstrated its capability in identifying a large amount of possible candidate variants in this study. By using very stringent criteria, we could identify four possible candidate genes with a putative role in cancer, however we have probably excluded several candidate variants in the filtering. Thus, the challenge is how to rank the variants in the candidate list to obtain an optimal balance between sensitivity and specificity.

In the search for a recessive syndrome, we found no cases of rare biallelic variants in any known cancer genes in our patients. We also searched the entire exome for more common autosomal recessive alleles and listed 16 possible candidate genes (Table 4) with six of them (*ATP10B*,* PKHD1*,* PTPRQ*,* UGGT2*,* MYH13*,* TFF3*) more likely than the others based on their MMAF <5% and observed frequency 20 times higher than the expected likelihood of occurring together. *ATP10B* is the catalytic part of a complex which catalyzes the hydrolysis of ATP coupled to the transport of aminophospholipids from the outer to the inner leaflet of the late endosomes (UniProt). It has been reported to be mutated somatically in 1.2% of all CRC in cBioportal (accessed 20181218), but its possible function in cancer is not yet known. *PKHD1* is known to cause autosomal recessive Polycystic Kidney Disease (MIM #606702). It encodes a protein which is important for correct mitotic spindle formation and function and inhibition leads to mitotic defects (Zhang et al., 2010). *PKHD1* is mutated in 5% of all CRC in cBioportal and in an analysis of 13,023 genes in 11 colorectal cases, *PKHD1* was ranked as the seventh most common somatically mutated gene (Sjoblom et al., 2006). However, there was a debate whether the finding reached statistical significance (Forrest & Cavet, 2007; Ward et al., 2011). *PTPRQ,* Protein tyrosine phosphatase receptor‐like type Q, was reported to be involved in phosphorylation/dephosphorylation signaling pathways and metastasis (Laczmanska et al., 2014, 2016; Sato et al., 2017). 1.2% of all CRC have somatic mutations in this gene (cBioportal, accessed 181218), however, as a suspected oncogene, this gene is a less likely candidate for an autosomal recessive predisposition gene. *UGGT2* is a uridine diphosphate‐glucose:glycoprotein glucosyltransferase (Takeda et al., 2014). It has no known function in cancer although 2.4% of all CRC carry somatic mutations in *UGGT2* (cBioportal). *MYH13* also has no known role in cancer, but somatic mutations in CRC are found in 2.3% (cBioportal). *TFF3*, Trefoil factor 3, is a secreted protein which stimulates cell migration and prevention of apoptosis, enabling repair of the intestinal mucosa. It was suggested to be a risk factor for early recurrence of CRC and to be involved in promoting lymph node metastasis (Huang, Li, Wang, & Zhang, 2013; Morito et al., 2015). although TFF3 is very rarely mutated in CRC tissue (0.1%, cBioportal). In all, the relevance of our detected possibly bi‐allelic variants is unknown. In summary, we have detected six putative candidate genes, of which five (*ATP10B*,* PKHD1*,* UGGT2*,* MYH13*,* TFF3)* may be candidate risk factor recessive genes for CRC.

Using the breast cancer cohort as a comparison to search for autosomal recessive genes may not be a good approach since there are genes that are predispose to both breast‐ and colorectal cancer. However, to look for possible compound heterozygous variants, we need a cohort where we have all the genotyping information. We have considered using genotyping data from public databases but there are a few issues. First, the number of samples with available genotyping information is far fewer than those that provide only allele frequency, due to data privacy. The second issue is about platform errors. Not all the variants we see in the analysis are from the samples. The errors can happen in any steps after DNA isolation: errors caused by contamination, by library preparation, by sequencing machines, or errors caused by computational tools. An advantage of our inhouse BRC cohort is that they are Swedish, the same population as the study group. Also, the DNA from the BRC cohort has been collected and processed in the same way as the study group. These advantages have eliminated the artifacts caused by the differences in populations and the difference in platforms.

The number of our proposed candidate truncating variants (10 variants in 51 cases) are slightly fewer than expected compared to those with similar study (26 of the 102 cases) (Adam et al., 2016), considering that our frequency cut‐off is less stringent (0.1% vs. 0.01%). This discrepancy can likely be explained by number of databases and number of sub‐populations used during filtering of rare variants. Adam et al. filtered their variants using 12 population databases including the seven ExAC cohorts. Our proposed candidate variants were required to have a MMAF <0.1% in 21 population databases. Among them, SweGen (Ameur et al., 2017) and our in‐house database of 249 Swedish samples played a major role in removing normal variants belonging to the Swedish population. The ALL population in ExAC and gnomAD (Lek et al., 2016), together with their other seven sub populations, even though they overlapped, contributed significantly with variants belonging to other populations. Databases of 200 Danish samples (Li et al., 2010) and 1000Genomes (ALL and NFE populations) (1000 Genomes Project Consortium et al., 2012) were also included to increase the sensitivity. This shows the importance of using population‐specific databases of normal variants in the filtering stage.

Our results (2/51 = 4% with a hereditary cancer syndrome: Cowden and *PMS2*‐Lynch syndrome) are comparable to others. For example Pearlman et al. showed that 2% of all CRC cases with onset before 50 years had a hereditary cancer syndrome other than Lynch syndrome (*APC, MUTYH, SMAD4*). They detected Lynch syndrome in 8%, especially in those with a family history (Pearlman et al., 2017). In our cohort, we selected patients with no family history, which largely excludes Lynch syndrome, although CRC caused by *PMS2‐*mutation with its low penetrance can occur in the absence of family history. As *PMS2* has multiple pseudogenes, pathogenic variants are often missed by WES and need to be manually analysed.

Simplex CRC cases are often caused by other mechanisms such as somatic mutations (Haraldsdottir et al., 2014; Mensenkamp et al., 2014) or epigenetic aberrations (Moreira et al., 2015). In the near future, patients with cancer will likely undergo paired tumor and germline testing in order to detect somatic aberrations that can guide therapy as part of genomic medicine initiatives across the Western world. As a bonus, both hereditary variants and somatic driver mutations can be identified in the same analysis. Until such pipelines are established, our results and those of others suggest that, in the absence of family history, or other suggestive features suggestive of hereditary syndromes such as polyposis or multiple tumors, genetic testing for gemline mutations in young CRC patients is of limited clinical benefit.

## 5 CONCLUSION

Whole exome sequencing in early onset non‐familial CRC patients only identified a causative germline mutation in two of 51 (4%). These patients had Cowden syndrome, that had not been diagnosed clinically or *PMS2*‐Lynch syndrome that was difficult to detect using WES. Our results suggest germline analysis using WES or broad gene panels in simplex cases with CRC at young age is of limited value in the clinic. In addition, we propose three candidate variants in known cancer susceptibility genes (*BMPR1A*,* BRIP1,* and *SRC*), up to three truncating variants in possibly novel cancer genes (*CLSPN* possibly *SEC24B*,* SSH2*), four missense variants in genes involved in cancer initiation or progression (*ACACA*,* NR2C2*,* INPP4A,* and *DIDO1*), and five candidate risk factor recessive genes (*ATP10B*,* PKHD1*,* UGGT2*,* MYH13*,* TFF3)*. Further studies are needed to find support for the pathogenicity of these variants in novel Mendelian or complex disease in early onset non‐familial CRC.

## CONFLICT OF INTEREST

The authors declare no conflict of interest.
